# Molecular Pathogenesis and Disease-modifying Therapies of Alzheimer’s Disease and Related Disorders

**DOI:** 10.31662/jmaj.2022-0079

**Published:** 2022-06-17

**Authors:** Takeshi Iwatsubo

**Affiliations:** 1 Department of Neuropathology, Graduate School of Medicine, The University of Tokyo, Tokyo, Japan; 2 National Institute of Neuroscience, National Center of Neurology and Psychiatry, Tokyo, Japan

**Keywords:** Alzheimer’s disease, dementia, amyloid β, disease-modifying therapy

## Abstract

The deposition of amyloid β (Aβ) peptides as senile plaques and tau as neurofibrillary changes causes the hallmark neuropathological lesions of Alzheimer’s disease (AD), which are implicated in its pathogenesis and deemed as the prime target for disease-modifying therapies (DMTs). Aβ is produced by sequential proteolytic cleavage by β- and γ-secretases. γ-Secretase, harboring presenilins (PS) as the catalytic center, forms the C-terminus of Aβ that determines its propensity to aggregate; missense mutations in PS genes cause familial AD by altering the preferred γ-secretase cleavage sites to increase the production of pathogenic Aβ42 species. Numerous DMTs for AD have been tested in clinical trials, some of which met the clinical endpoints, whereas others, especially those conducted in dementia stages, have failed, underscoring the needs for early intervention. Notably, positive outcomes of recent trials for anti-Aβ antibody drugs have depended largely on molecular imaging and fluid biomarkers, underscoring the needs of markers that surrogate the clinical and pathophysiological progression of AD. Longitudinal observational studies as represented by the AD Neuroimaging Initiative (ADNI) in North America, as well as the Japanese ADNI (J-ADNI), have contributed greatly toward the goal of very early treatment at the prodromal and preclinical AD stages by delineating the early natural course of AD and facilitating the development of biomarkers. It has been demonstrated that the clinical and biomarker profiles of prodromal AD in J-ADNI were remarkably similar to those in the North American ADNI, supporting the harmonization of global clinical trials. These clinical studies in Japan, accelerated by the development and implementation of biomarkers, will pave the way toward the development of AD therapies targeting its very early stages.

## 1. Introduction

Alzheimer’s disease (AD) is the most frequent cause of neurodegenerative dementia in the elderly. Currently, the number of individuals with dementia in Japan exceeds 6 million, among which patients with AD are estimated to comprise ~3 million. The elucidation of the pathogenesis of AD and the clinical implementation of mechanism-based therapies (i.e., disease-modifying therapy [DMT]) are urgently needed.

Pathologically, AD is characterized by neuronal loss in the cerebral cortex, accumulation of neurofibrillary tangles within neurons, and the formation of senile plaques. Neurofibrillary tangles are formed by abnormal fibrils composed of tau protein and are associated with neuronal cell death. Senile plaques are hallmark lesions of AD brains composed of extracellular accumulation of amyloid fibrils associated with dystrophic neurites and activated glial cells. Senile plaques are composed of amyloid β (Aβ) peptides, highly specific to AD, and are the earliest pathological changes that appear during the progression of AD pathology. In particular, the fact that the pathogenic gene mutations causative to familial AD enhance Aβ aggregation provided strong evidence supporting the “β-amyloid hypothesis,” which postulates Aβ as the pathogenic molecule causative to AD.

## 2. Pathological Analyses of Brain Amyloid in AD

**Figure 1.**
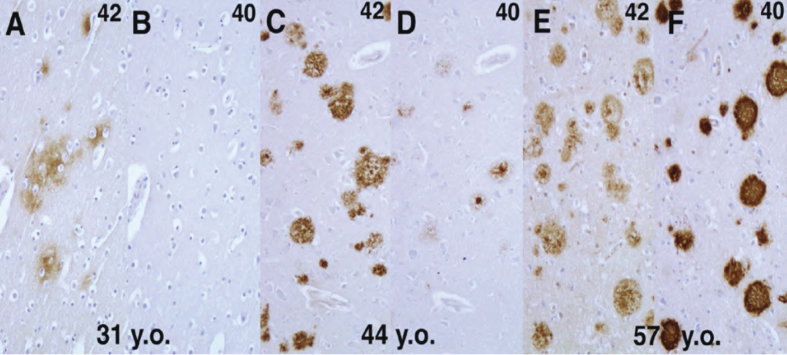
Aβ42 deposition precedes that of Aβ40 in the progression of AD changes in Down syndrome brains. Frontal cortices from autopsied brains from patients with Down syndrome who died at the ages of 31 (A, B), 44 (C, D), and 57 years (E, F) were immunostained with Aβ42- (A, C, E) and Aβ40-specific monoclonal antibodies.

Masters and colleagues found that senile plaques are composed of Aβ peptides with a variable carboxy (C)-terminus, including Aβ40 species terminating at valine 40, and Aβ42 extending to alanine 42 ^(1)^. *In vitro* experiments by Lansbury’s group suggested that the C-terminal length of Aβ has a pathogenic significance, as the Aβ42 species facilitates the formation of “aggregation nuclei” of Aβ, which in turn promotes the formation of amyloid fibrils ^(2)^. We were interested in the relationship between Aβ40 and Aβ42 species during the progression of human brain amyloidosis, and have shown by immunohistochemical analyses of Down’s syndrome brains, which develop early signs of amyloid accumulation in the form of diffuse senile plaques in youth and reproduce the pathological process of AD with aging, using Aβ40 and Aβ42-specific antibodies, that Aβ42 begins to accumulate first, and Aβ40 starts to deposit later in the progression of human AD pathology (Figure 1). These findings were intriguing in showing that differences in the physical properties (i.e., aggregability) based on minute differences in the primary sequence of the deposited proteins may be reflected in the process of amyloid accumulation ^(3), (4)^.

## 3. Analyses of Familial AD Genes-pathogenic Roles of APP and PSs

Definitive evidence supporting the causal significance of Aβ came from genetic analyses of familial AD. The Aβ precursor protein (APP) gene was the first pathogenic gene identified in the autosomal dominantly inherited familial AD (FAD). Suzuki and Younkin found that the London-type APP mutations cause a shift of γ-secretase cleavage in a way to increase Aβ42 production, the latter being more prone to aggregation ^(5)^. We also confirmed an increased Aβ42 deposition ratio by immunohistochemical analyses of autopsied brains of Japanese patients with FAD harboring the London-type mutation ^(3)^. Thus, the pathogenic effects of APP mutations causing FAD converged to the abnormalities at the level of production that accelerate Aβ aggregation ^(5), (6)^.

Furthermore, presenilin 1 (PS1; *PSEN1*) and the homologous PS2 (*PSEN1*) genes were identified as the major FAD-causing genes on chromosomes 14 and 1, respectively. *PSEN1* and *PSEN2* both encode homologous 9-transmembrane proteins, although their normal function was initially elusive. We have expressed the Volga German mutant PS2 in cultured cells and found an increased secretion of Aβ42 ^(7)^. The same effect was reported by other investigators for the FAD mutant PS1 as well, indicating that mutations in *PSEN1* and *PSEN2*, like the APP mutation, have the identical effect of increasing the production of highly aggregable Aβ42 species.

## 4. Nature and Formation Process of the γ-secretase Complex

It was not until 2000 that Li et al. found that the transition state-analog protease inhibitors theoretically bind to the active center of the aspartyl protease target PS1, demonstrating that the PS1 polypeptide represents the active subunit of γ-secretase ^(8)^. However, it has been well known that the PS polypeptide alone does not exhibit γ-secretase activity; this led to an assumption that a high-molecular-weight complex containing protein cofactors other than PS might be the *bona fide* γ-secretase in its active form ^(9)^. The introduction of the RNA interference method in *Drosophila* cells gave clear-cut answers ^(10)^: suppression of nicastrin (NCT), a single transmembrane protein initially identified as a cofactor for γ-secretase, reduced the γ-secretase activity, whereas coexpression of PS and NCT did not increase the enzymatic activity, strongly suggesting the presence of additional cofactor proteins. The third and fourth cofactors were identified by genetic screening: PS1 knockout mice present developmental abnormality and are embryonic lethal due to impaired cleavage and activation of Notch by γ-secretase; analysis of loss-of-function mutant of Notch in *Caenorhabditis elegans* revealed the presence of a seven-transmembrane protein APH-1 and a two-transmembrane protein PEN-2. However, the role of either of the cofactor proteins in the formation and activation of the γ-secretase complex remained unknown. In addition, a critical question remained whether these four factors, including PS, are sufficient for the full activity of γ-secretase.

**Figure 2.**
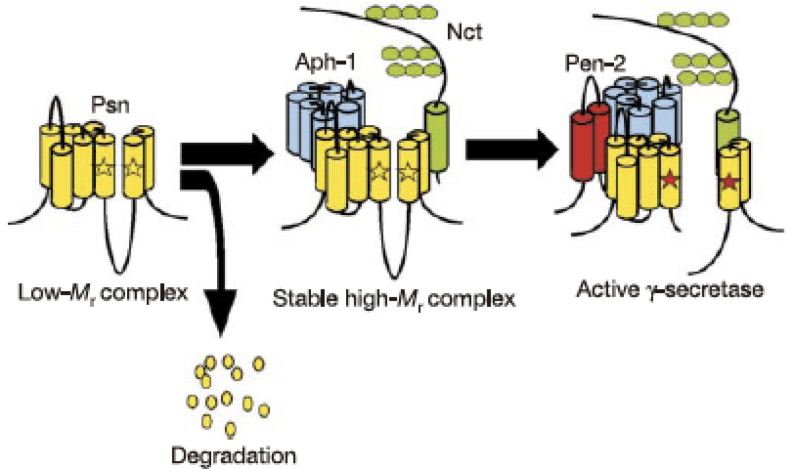
Schematic depiction of the process of γ-secretase complex formation.

We further investigated the process of formation and activation of the γ-secretase complex by overexpression experiments ^(10)^; in the presence of endogenous PS, expression of NCT alone did not alter the endogenous PS, whereas additional expression of APH-1 did not change the level of PS fragments but increased that of the PS holoprotein in a stable high-molecular-weight complex, as observed with the fragment form, although without an increase in the γ-secretase activity. Next, we found that the fragment forms of PS disappeared on knockdown of PEN-2, and the PS holoprotein in the high-molecular-weight complex was stabilized, exactly as observed with the coexpression of APH-1 and NCT. Eventually, coexpression of APH-1, NCT, and PEN-2 resulted in an increase in PS fragments accompanied by elevated γ-secretase activity. Collectively, these results suggest that APH-1 acts as a stabilizing factor for the γ-secretase complex, whereas PEN-2 is responsible for its maturation and confers the γ-secretase with proteolytic activity ^(11)^. Thus, it was concluded that PS, NCT, APH-1, and PEN-2 are necessary and sufficient to constitute the γ-secretase complex (Figure 2). Thus, γ-secretase has attracted enormous attention as a new class of protease that is responsible for intramembrane proteolysis.

## 5. Structural and Functional Analyses of the γ-secretase Complex

The elucidation of the question as to how γ-secretase brings in water molecules into the hydrophobic lipid environment to hydrolyze the transmembrane sequences of its substrates is important for the understanding of the mechanism of “intramembrane proteolysis” as well as for the development of γ-secretase inhibitors as AD drugs. However, γ-secretase is a huge complex composed of multiple membrane proteins, making it difficult to apply conventional analytical methods, for example, protein crystallography.

Our γ-secretase structural analysis project, headed by Dr. Taisuke Tomita, set out to conduct a series of functional and structural analyses of the PS1 protein, the catalytic center subunit of γ-secretase, applying the substituted cysteine accessibility method utilizing cysteine chemistry techniques that label cysteine residues exposed to hydrophilic milieu, and analyzed the structure of PS1 around the catalytic aspartate residues at its active center. We demonstrated that the sixth and seventh transmembrane domains form a hydrophilic “catalytic center pore” structure within the lipid bilayer ^(12)^, and that the PAL motif and the ninth transmembrane domain of PS1 contribute to the formation of the catalytic pore ^(13)^. We also showed that transmembrane domain 1 on the N-terminal side of PS1 directly faces the catalytic pore structure and functions as a subsite (i.e., a substrate-binding site distinct from the catalytic site) ^(14)^. Our results based on the substituted cysteine accessibility method of PS1 were proven to be in good agreement with the recent results of crystallographic and cryo-electron microscopy analyses of γ-secretase. Thus, the functional and structural information on PS has contributed greatly to the understanding of the novel concept of intramembrane proteolysis as well as to the development of γ-secretase inhibitors and modifiers.

## 6. Identification of Phosphorylated α-synuclein in LBs and Involvement of LRRK2 in Parkinson’s Disease

**Figure 3.**
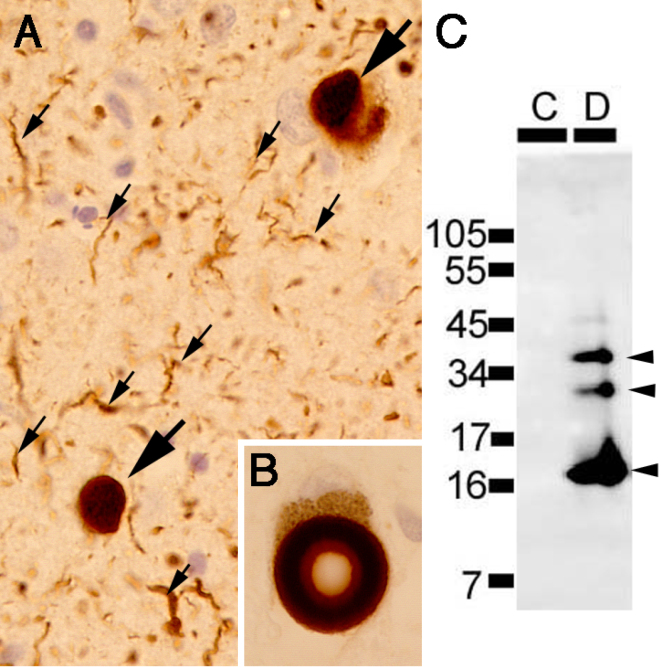
Histopathological and biochemical features of a-synuclein deposited in PD and DLB brains. Immunohistochemistry of cortical-type Lewy bodies (large arrows) and Lewy neurites (small arrows) in DLB cortices (A) and brain stem-type Lewy body in the substantia nigra of PD brain (B) labeled by a phosphoserine 129-specific anti-α-synuclein antibody. C: Immunoblot analysis of sarkosyl-insoluble fractions from control (C) and DLB (D) cortices with a phosphoserine 129-specific anti-α-synuclein antibody (C).

The tremendous impact of the identification of brain-accumulated proteins on the elucidation of the pathogenesis of AD led us to set out to identify the components of Lewy bodies (LBs), hallmark intra-neuronal inclusions diagnostic of Parkinson’s disease (PD) and dementia with Lewy bodies (DLB). We first established a method to isolate and purify cortical-type LBs, exhibiting a spherical shape with a diameter of ~10 μm, using immunofluorescence staining for known components (e.g., ubiquitin) by cell sorters ^(15)^. We raised monoclonal antibodies against the purified LBs as immunogen and obtained a monoclonal antibody LB509 by immunostaining screening of the crude LB fraction, which recognized a ~15-kDa protein on immunoblots of the soluble fraction of human brains. At the same time, Polymeropoulos of the National Institute of Health of the US reported a mutation in the α-synuclein gene in a pedigree of autosomal dominantly inherited familial PD, which led us to demonstrate that the antigen in LB recognized by LB509 was α-synuclein ^(16)^. We further pursued to identify posttranslational modifications specific to accumulated α-synuclein by mass spectrometric analyses of insoluble α-synuclein recovered from DLB brains and found that the Ser129 residue of α-synuclein was highly and specifically phosphorylated (Figure 3) ^(17)^. α-Synuclein is a soluble protein abundant in the presynaptic termini of the brain, and our findings, together with the genetic evidence derived from familial PD studies, consolidated the concept that conformational alterations of α-synuclein lead to its aggregation within neurons and eventually neuronal death. Clarification of the mechanisms of pathogenic proteins in various neurodegenerative diseases, including synucleinopathies and tauopathies ^(18), (19)^, as well as elucidation of the basic mechanisms underlying neurodegeneration, for example, excessive lysosomal stress in relation to the function of LRRK2 linked to familial PD ^(20)^, will greatly facilitate the development of therapeutic strategies against PD.

## 7. From Bench Side to Clinic: Contribution of J-ADNI to Development of DMTs of AD

Efforts to develop DMTs that directly act on the mechanism of AD pathophysiology and aim to change the disease course started at the global level at the beginning of the 21st century. However, early clinical trials for AD in the dementia stage (AD dementia) did not demonstrate sufficient efficacy, which led to the consensus that therapeutic intervention by DMTs should be applied at earlier stages. DMT trials in the mild cognitive impairment (MCI) stage, a mildly symptomatic stage preceding dementia, were expected to face difficulties in evaluating the clinical efficacy because of the slow rate of progression at earlier stages. To overcome this obstacle, the AD Neuroimaging Initiative (ADNI) was launched in the United States in 2004 ^(21)^, with the aim of accurately evaluating the progression of AD and enabling precise evaluation of drug efficacy in DMT trials at an early stage, such as the MCI stage, by adopting neuroimaging (e.g., magnetic resonance imaging and positron emission tomography [PET] scans) and fluid biomarkers. In Japan, however, the development and implementation of precise diagnostic techniques in AD, as well as establishing a database on MCI and the very early stage of AD, has been left behind, despite significant early efforts in AD clinical research, including those to develop blood markers ^(22), (23)^.

**Figure 4.**
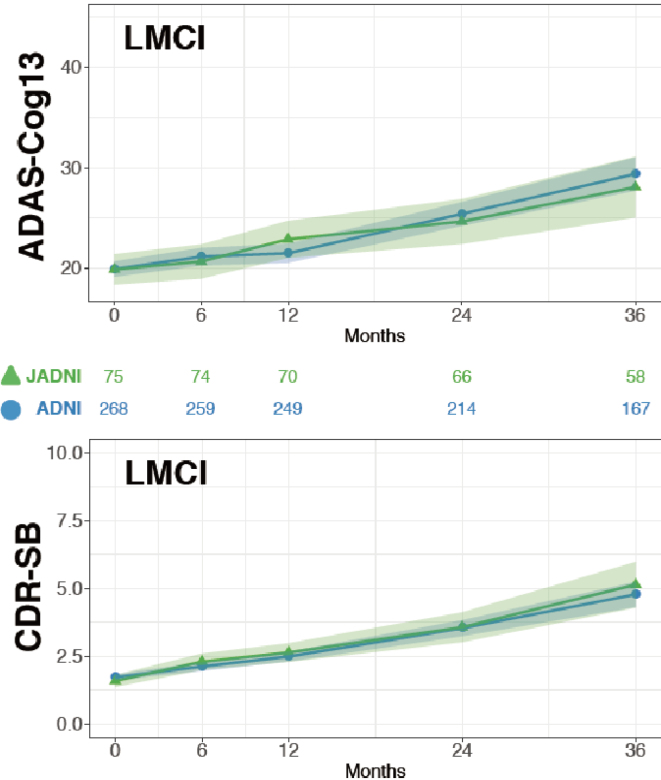
Comparison of cognitive changes (Alzheimer's Disease Assessment Scale-Cognitive Subscale: ADAS-Cog13; upper panel) and clinical progression (Clinical Dementia Rating-sum of boxes: CDR-SB; lower panel) in late MCI due to AD (LMCI) in the J-ADNI (green lines) and the North American ADNI (blue lines); cited from Iwatsubo et al. ^(23)^.

**Figure 5.**
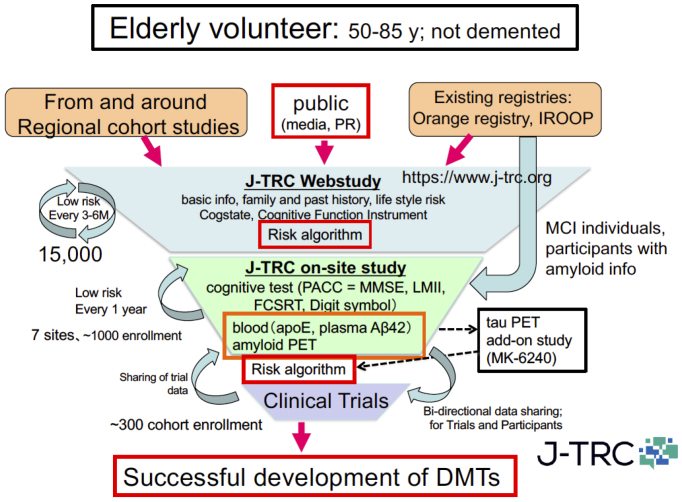
Overview of the Japanese trial-ready cohort (J-TRC) for preclinical and prodromal AD.

To challenge this situation, the Japanese ADNI (J-ADNI) study was started in 2007, bringing together clinical researchers and brain imaging and biomarker experts from 38 representative AD clinical research institutions across Japan. A total of 537 cases (234 late MCI, 149 mild AD dementia, and 154 healthy elderly individuals) were studied for 24-36 months, and a detailed longitudinal study of the natural history of these different early AD/dementia stages was completed ^(24)^. Based on the J-ADNI data, it was first demonstrated that there is a high degree of cross-racial similarity between the Japanese and Americans in the progression profiles of amnestic MCI that corresponds to the early stage of AD (Figure 4). The J-ADNI study enabled the nationwide implementation of amyloid PET, biomarker sample handling, and cognitive function assessment harmonized at the global standard level, making it possible to precisely assess subtle changes in cognitive and pathophysiological progression under a clinical trial setting of DMTs. The success of the J-ADNI in Japan accelerated the clinical development of DMTs that target pathogenic factors of AD, for example, Aβ and tau, and enabled global clinical trials for MCI in Japan. Further efforts to establish trial-ready cohorts for preclinical and prodromal AD (the J-TRC study) are ongoing, which will expand the spectrum of early AD trials in a way to cover the earliest asymptomatic stage of AD (Figure 5) ^(25)^.

Recently, significant progress in DMT development in the early AD (i.e., MCI to mild dementia stage) was achieved with the completion of two global phase III studies of aducanumab, an anti-Aβ antibody drug ^(26)^, and its accelerated approval by the US FDA. The technology and network established by the J-ADNI was essential to the completion of the aducanumab trial in Japan that recruited 221 participants with early AD, and will strongly support the upcoming series of global DMT trials. The sharing of information and data among academia, clinical site physicians, pharmaceutical companies, PET imaging facilities, regulators and government, and, most importantly, study participants is vital to the successful development of clinically meaningful medications. The development of DMT for AD and other related disorders in the near future will greatly contribute to the relief of people suffering from dementia.

## Article Information

This article is based on the study, which received the Medical Award of The Japan Medical Association in 2021.

### Conflicts of Interest

None
